# Unveiling patterns: A cross-sectional analysis of drug prevalence among secondary school students in Douala, Cameroon

**DOI:** 10.1371/journal.pmen.0000016

**Published:** 2024-06-04

**Authors:** Tekuh Achu Kingsley, Asongalem Emmanuel Acha, Njunda Anna Longdoh, Nsagha Dickson Shey

**Affiliations:** 1 Department of Public Health and Hygiene, Faculty of Health Sciences University of Buea, Buea, Cameroon; 2 Department of Biomedical Sciences, Faculty of Health Sciences University of Buea, Buea, Cameroon; GC Women University Sialkot, PAKISTAN

## Abstract

The escalating prevalence of psychoactive substance use (Pa SU) presents a significant concern in the African context, particularly among schooling adolescents, leading to potential physical and mental health complications, making substance use a giant monster for every developing society. To address this, the study focused on determining the prevalence and understanding the factors associated with psychoactive substance (Pa SU) among secondary school students in the Douala III and Douala IV districts. A cross-sectional study was conducted from January to March 2023, engaging students in secondary schools in the Douala III and Douala IV districts. Utilizing a self-administered questionnaire, comprehensive data on student sociodemographic, parental social information, and patterns of psychoactive substance use were collected. Statistical analyses, performed using SPSS, explored associated factors, with statistical significance set at p < 0.05. Enrolling 1054 students, the study reflected a male-to-female sex ratio of 3:2, with an average age of 15.29 ± 1.9 years (range: 12 to 20 years). Psychoactive substance life tune use (Pa SU) experimentation prevalence reached 91.0%, while current consumption was noted at 42.8%, exhibiting a male predominance. Alcohol emerged as the most frequently consumed substance, succeeded by caffeine and nicotine. Cannabis stood as the sole illicit substance, with tramadol being the only psychotropic medication used without medical guidance. Male gender (AOR = 1.58; CI: 1.07–2.34; p = 0.022), age > 16 years (AOR = 2.94; CI: 2.02–4.27; p < 0.001), the presence of a family member using psychoactive substances at home (AOR = 3.80; CI: 2.61–5.53; p < 0.001), and the presence of a friend using psychoactive substances in the surroundings (AOR = 32.92; CI: 22.02–49.20; p < 0.001) were independently identified as risk factors associated with current PaSU among students. This study provides valuable insights into the prevalence and associated factors of psychoactive substance use among secondary school students in Douala, Cameroon. The results underscore the urgent need for targeted interventions and parental awareness programs to mitigate the impact of psychoactive substance use on adolescents.

## Introduction

The alarming rise in drug use (psychoactive substance use or “alterno-substance use”) among today’s youth has ignited a global health crisis. From bustling metropolises to remote corners of the globe, adolescents are increasingly drawn into this perilous web, influenced by a myriad of factors. Worse still among secondary school students, the situation is dire, with a plethora of influences driving their engagement in substance use. psychoactive substance use is therefore a giant societal monster that affects all ages, races, genders, professions, and no geolocation is free from it. Although psychoactive substance use (alterno-substance use) is mostly individualized, its repercussions are felt mainly at the societal level.

Understanding the multifaceted nature of adolescent substance use is crucial for developing targeted prevention and intervention programs that address the underlying determinants of drug use among young individuals. Finding from existing literatures emphasizes the urgency of implementing evidence-based interventions and policies to mitigate the prevalence of drug use among young adolescents.

## Background

### Prevalence rate and factors contributing to substance use

A comprehensive understanding of substance use among secondary school students can be achieved through prevalent studies that explore various factors associated with substance abuse, especially in a country like Cameroon with very limited research on adolescent substance use. However, the geo-localised availability of finding on adolescent related substance use in neighbouring countries and other Africa countries with contextualized similarity like Cameroon, revealed significant interest on the necessity to comprehend the prevalence of substance use and its determinants. Nonetheless, a study conducted in Cameroon found notable statistics on psychoactive substance abuse. Legal commercialized substances like alcohol were in highest demand (45.9%), followed by tobacco (28.8%), and volatiles (11.5%). Additionally, illegal substance use, particularly cannabis (10.3%), was most reported among street children. This pattern was especially prevalent among males in major urban cities in Cameroon [1]. Although the study was not centre around secondary school student it sheds some light on the prevalence and risky behaviours around the use of psychoactive substance in Cameroon [1].Similarly, a study on the prevalence and associated factors of substance use among students in Nigeria revealed several findings. For instance, over-the-counter and socially acceptable substances such as caffeine (kolanuts and coffee) and prescription medicines had varying lifetime use prevalence rates. Alcohol and tobacco had lifetime use prevalence rates of 9.2% and 5.2%, respectively [2]. Moreover, the abuse of illicit substances was common among secondary school students in urban settings in Nigeria [2]. This highlights the continued major risk behaviour among youth and its consequent physical and mental health complications [2]. As such, the reasons advanced by existing literature for substance use includes: biological factors such as genetic predisposition to alcohol abuse, trauma, the presence of psychiatric disorders such as depression, anxiety or stress; social factors such as bullying and environmental factors such as community disorganization has been associated with drug use (alterno-substance use) among this demographic [3]. Additionally, the need to show off one’s status in society has been identified as a motive for drug abuse, with students indulging in substances such as cocaine, tramadol, and heroin to display their social class with social consequences linked to disciplinary problems and witnessing, carrying, and use of drugs and dangerous weapons [4, 5]. Moreover, the influence of family environment and parental support were also found to play a role in the prevalence of illicit drug use among students in secondary schools and associated behavioural risks among secondary school students [6]. Similarly, in another study exploring the knowledge and indulgence in substance abuse among adolescents in Anambra state, South-East Nigeria, it provided insights into the multi-domain factors contributing to substance use among adolescents such as: such as community norms endorsing substance use, family history of alcohol and substance use, siblings’ substance use, poor academic performance, low perceived risk of substances, and friends’ substance use were positively associated with adolescent substance use [7].

Studying drug prevalence among secondary school students in metropolis like Douala, Cameroon is of paramount importance due to its potential impact on the students’ well-being and academic performance. Research has shown that the living environment of young people is contaminated with various psychoactive substances, emphasizing the need to understand the prevalence and factors associated with substance abuse among adolescents [8]. Another study conducted in Cameroon among medical and nursing students in Cameroon show that they are likely to experience mental health problems due to the stressful nature of their studies, highlighting the importance of addressing substance abuse among students [9]. Furthermore, integrated interventions in school settings have been shown to have substantial impacts on important health risk behaviours, emphasizing the potential for school-based interventions to address substance abuse among students [10]. Understanding the prevalence of drug use among secondary school students in Douala is crucial for developing targeted prevention programs and promoting the overall well-being of students in the region.

### Impacts on academic performance and mental health

It is evidence that substance abuse among adolescents, particularly those in secondary school, is a global concern with far-reaching implications for mental health and societal well-being, with various studies highlighting the prevalence of substance abuse among youth in various regions [11]. The use of psychoactive substances among adolescents has been associated with mental illness, including depression and posttraumatic stress disorder [11–13]. More also, the relationship between substance abuse and risky behaviours, such as premarital sex and high-risk sexual behaviours and viral infections such as hepatitis B and HIV underscores the complex interplay of factors influencing adolescent decision-making [14]. The impact of substance abuse on academic performance and discipline among secondary school students has also been a subject of investigation, with studies revealing a positive correlation between substance abuse and academic achievement [15–17]. Further evidence on environmental and social factors, such as peer influence, availability and affordability of substances, and parental attitudes towards substance use, are further identified significant contributors to adolescent substance abuse. Additionally, the association between childhood abuse and adolescent substance misuse highlights the role of traumatic experiences in shaping maladaptive coping mechanisms, including drug use [18]. The influence of slum living and poverty on substance addiction among students further emphasizes the multifaceted nature of this issue [17]. The variability in drug use prevalence across school districts within such locality underscores the need for targeted interventions that consider local contextual factors influencing substance abuse among adolescents [19]. Moreover, the high-risk behaviours of street-involved youth, including intravenous drug use, highlight the vulnerability of marginalized populations to substance abuse and its associated health risks [20, 21].

### Challenges and opportunities in education systems

Substance abuse among adolescents in Cameroon, particularly those in secondary school, is a pressing public health concern with profound implications for individual well-being and societal development. Understanding the local factors influencing drug prevalence and its public health consequences is crucial for developing effective intervention strategies tailored to the specific needs of Cameroonian adolescents. Studies from diverse cultural contexts have highlighted the significance of understanding the factors associated with substance abuse among adolescents. For instance, a research conducted in Nigeria emphasized the interference of substance abuse with normal adolescent development, underscoring the importance of studying school populations to comprehend the dynamics associated with substance abuse in this age group [22]. Similarly, a study in South Africa also reveals the challenges faced by parents with adolescents abusing substances, emphasizing the need for professional assistance and support to promote, maintain, and restore their mental health [11]. Furthermore, another research in Malaysia identified risk factors for illicit drug use among adolescents, providing valuable insights for the development of intervention programs to combat substance abuse at the school level [23].

The secondary education system in Cameroon consists of both the Francophone and Anglophone subsystems, each influenced by the French and British educational systems, respectively [24]. In an urbanized areas like Douala, the dual nature of education presents both challenges and opportunities. This is due to the diverse socio-economic backgrounds, cultural influences, and resource accessibility among students. Understanding the prevalence of drug use among the diverse population of adolescents in Douala’s secondary education system is crucial. It can have significant impacts on academic performance, mental health, and overall societal well-being.

By understanding the local context and its implications for drug prevalence, targeted interventions and policies can be developed to support the well-being and academic success of secondary school students in different regions of Cameroon.

## Methods

### Study design and study area

A cross-sectional study as part of a larger study entitled: “Impact of health education on the use of psychoactive substance among secondary school students in Douala, Cameroon”. Was conducted in 2022/2023 academic year for 3months (January to March 2023) in Douala, Cameroon, focusing on 08 classes from two bilingual-mix gender public government schools, which are all located in two urbanized district which are the Douala IV and the Douala III District respectively in Wouri Division. These institutions of learning were randomly selected for the study which was geared to determine the prevalence and patterns of drug use, while identify the determinants, academic performance, predictors of drug use and overall well-being of secondary school students in Douala, Cameroon.

Douala, is the largest city in Cameroon and serves as the economic capital of the country. It is a bustling urban centre with a diverse population and a range of socio-economic backgrounds [25]. The study population comprised secondary school students from these government schools, representing a diverse demographic of adolescents. The choice of these schools provided a representative sample of students from different socio-economic and cultural backgrounds, allowing for a comprehensive assessment of drug prevalence and associated factors, with the aim to investigate the prevalence and patterns of drug use among secondary school students in Douala, Cameroon. The cross-sectional design was chosen for its suitability in capturing a snapshot of drug prevalence among secondary school students in Douala and as such, to provide valuable insights into the prevalence, patterns, and determinants of substance abuse among students, informing targeted interventions and policies to address substance abuse in educational settings.

### Study population and inclusion criteria

The study was conducted over a period of three months, commencing on January 1, 2023, and concluding on March 31, 2023. The target population comprised secondary school students enrolled in form two, form three, form four, and lower sixth classes in the anglophone section, as well as sixième, cinquième, quatrème, and seconde classes in the francophone subsystem of education within the urban districts of Douala III and Douala IV in the Wouri Division. Inclusion criteria encompassed students in the first and second cycles in a bilingual government school who provided informed assent to participate in the study. Exclusion criteria were defined as students who explicitly declined to participate in the survey, students in examination classes, and students without informed assent to participate in the study.

### Sampling size determination

We utilized the single population proportion formula for an infinite population, the minimum sample size of 283 ~ 300 students was obtained using a national prevalence of drug use as reported by the Cameroon’s Anti-drug National Committee (CNLD) of the Ministry of public health [26].


$$\eta =\frac{{ʑ}^{2}pq}{{ę}^{2}}$$

Where:

Ƞ = sample size

ʑ^₂^ = 1.96 at 95% Confidence interval

*p = anticipated prevalence of 21% (CNLD, Ministry of public health Cameroon)*

*q = (1-p)*

ę^₂^ = 0.05 that is 5% margin of error

Therefore, the calculated sample size is approximately 256.92. Since the sample size should be a whole number, we round up to ensure an adequate sample size. So, the revised sample size is 257.

To account for a non-response rate, the sample size was adjusted considering a 10% non-response rate: Non-Response Rate (10%) = 10% of 257 = 25.70

Adjusted Sample Size: 257+25.70 and rounding up to the nearest whole number:

Ƞ = **283** as minimum sample size

### Sampling procedure

The sampling methodology implemented in this study employed a two-stage stratified cluster sampling strategy to ensure the accurate representation of students within the two administrative districts [27, 28]. During the initial stage, the sampling frame was derived from the list of operational schools in the district for the academic year 2021–2022, as supplied by the Departmental Delegation of Secondary Education of Wouri/Regional Delegation for secondary education for the Littoral.

Stratification was based on the fact that all schools in the frame were public and bilingual in Douala III and Douala IV. This resulted in the identification of two distinct strata: public bilingual schools in Douala III and public bilingual schools in Douala IV. Following this, one schools were randomly selected within each stratum. In the subsequent clustering stage, the minimal sample size was apportioned proportionally based on the total number of students in each stratum. This count was subsequently divided by the number of first and second-cycle classrooms in each stratum for the anglophone and later for the francophone section of the institution to ascertain the quantity of classrooms to be chosen per school and per stratum. The selected classrooms were then randomly designated within each school participating in the study. Every student within the chosen classrooms participated willingly in the study, so as to careful consider any ethical issues thereby, ensuring that all students were included in the survey to guarantee a comprehensive representation across different groups or categories (strata). Special attention was given to obtaining informed assent from both students. Confidentiality measures were implemented to safeguard the privacy of participants, and the survey was conducted in a manner that respected the voluntary nature of their participation.

### Study variables and pre-testing

The study utilized the United Nations Office on Drugs and Crime (UNODC) recommended questionnaire for school-based surveys to collect data on drug consumption among students [29]. The questionnaire was modified to fit the study’s context, with adjustments made to the socio-demographic information section and the consumption-related sections. A pre-test was conducted in a selected secondary school to refine the questionnaire before data collection, ensuring its appropriate contextualization and relevance [30]. The study focused on variables such as sociodemographic information and consumption of various substances, including alcohol, tobacco, cannabis, and prescription medications like tramadol [31]. The study also considered non-response bias in survey estimates of alcohol consumption and its association with harm, highlighting the importance of accurate reporting in population surveys [32]. The variables studied included sociodemographic factors such as gender, age, religion, type of attended institution, and educational levels of parents or guardians [31].More also, the study examined variables related to the consumption of various substances, including the frequency of consumption during different time frames and circumstances of substance consumption, such as the age of first substance consumption and the presence of substance users in the family and among friends, the association between personality traits and social preferences, focusing on the characterization of social consumers based on their personalities and sociodemographic variables [33].

### Data collection

The statistical data underwent coding and analysis using SPSS software version 20. Descriptive statistics were employed, encompassing frequencies and proportions for categorical data, and means with standard deviations for continuous data. The comparison of categorical variables (proportions) was conducted utilizing the chi-square test to explore the factors associated with substance use. Initially, univariate analysis was performed to estimate crude odds ratios and their 95% confidence intervals (95% CI). Subsequently, variables that exhibited significant associations in the univariate analysis were included in multivariate models using a backward elimination procedure. A p-value less than 0.05 was deemed to be statistically significant.

### Ethical considerations

Ethical approval reference no. 2022/1846-05/UB/SG/IRB/FHS for the study was obtained from the Faculty of Health Sciences Institutional Review Board at the University of Buea. Administrative authorization was sought from the Littoral Divisional Delegation of secondary education (Reference no. 27/21/ACR/MINSEC/DRES-LT/DDESS-W). Informed written and signed parental assent was obtained from study participants before inclusion in the study, however, participation was voluntary with participants being able to withdraw from the study at any time. The study ensured that participants identity were anonymous as a strict degree of confidentiality was maintained during the study period and later during the dissimilation of results.

## Results

### Sociodemographic

The sociodemographic characteristics of the students were comprehensively analysed to provide a detailed overview of the study population as presented in Table 1 below, indicated that the sample consisted of 1054 students. And the distribution of the study population across various demographic and socioeconomic variables was examined.

**Table 1:** Categorical variables showing the sociodemographic characteristics of 1054 participants.

Variable	Pool n (%)	95% CI
**Sex**		
Male	635(60.2)	57.3–63.2
Female	419(39.8)	36.8–42.7
**Age (years)**		
Mean ± SD	15.29 ± 1.94	
Minimum	12	
Maximum	20	
**Age Groups**		
< 14 years	135(12.8)	10.8–14.8
14–17 years	883(83.8)	81.6–86.0
18–20 years	36(3.4)	2.3–4.5
**Subsystem of education**		
Anglophones	414(39.3)	36.4–42.2
Francophones	640(60.7)	57.8–63.6
**Education level**		
ordinary level	713(67.6)	64.8–70.5
advanced level	341(32.4)	29.6–35.2
**Religion**		
Catholic	727(69.0)	66.2–71.8
Protestant	231(21.9)	19.4–24.4
Muslim	18(1.7)	0.9–2.5
Other	66(6.3)	4.8–7.8
None	12(1.1)	0.5–1.7
**Daily pocket allowance**	
200	482(45.7)	42.7–48.7
300	294(27.9)	25.2–30.6
400	167(15.8)	13.6–18.0
500	68(6.5)	5.0–8.0
>500	43(4.1)	2.9–5.3

SD: standard deviation

The results revealed that the majority of the participants were male students, accounting for 60.2% (n = 635, 95% CI: 57.3–63.2), while females students constituted 39.8% (n = 419, 95% CI: 36.8–42.7) of the sample. The mean age of the participants was 15.29 years, with a standard deviation of 1.94. The age distribution indicated that the majority of the participants fell within the age group of 14–17 years, representing 83.8% (n = 883, 95% CI: 81.6–86.0), followed by those aged below 14 years (12.8%, n = 135, 95% CI: 10.8–14.8) and 18–20 years (3.4%, n = 36, 95% CI: 2.3–4.5). Regarding the subsystem of education, 60.7% (n = 640, 95% CI: 57.8–63.6) of the participants were Francophones, while 39.3% (n = 414, 95% CI: 36.4–42.2) were Anglophones. In terms of education level, the majority of the participants were student in the first cycle that is ordinary level cycle of education, accounting for 67.6% (n = 713, 95% CI: 64.8–70.5), while 32.4% (n = 341, 95% CI: 29.6–35.2) were 2^nd^ cycle student that is advanced level cycle of education. The distribution of participants based on religion revealed that the majority identified as Catholic (69.0%, n = 727, 95% CI: 66.2–71.8), followed by Protestant (21.9%, n = 231, 95% CI: 19.4–24.4), Muslim (1.7%, n = 18, 95% CI: 0.9–2.5), and other religions (6.3%, n = 66, 95% CI: 4.8–7.8). These findings provide valuable insights into the demographic and socioeconomic characteristics of the study population, which are essential for understanding the context of the research and interpreting the study findings.

### Prevalence from last drug use (alterno-substance use) by type

Table 2 above shows the various prevalence of different alterno-substance use (drug use) among secondary school student. The drugs commonly in used included alcohol, nicotine, cannabis, prescription medication (tramadol and diazepam), and caffeine. With regard to the period of last use. The data were stratified by gender and includes the chi-square test and p-values for each drug and period of use. Alcohol was the highly reported used drug by students with 90.2% of males and 89.5% of females indicating lifetime alcohol consumption. Additionally, a significant proportion of students reported alcohol use in the last 6 months, with 79.5% of males and 66.6% of females acknowledging consumption. The chi-square test indicated a statistically significant difference in the last 6 months alcohol use between males and females students (χ2 = 10.520, p = 0.005). Furthermore, the data showed that a substantial number of students had used alcohol in the last 30 days, with 48.0% of males and 32.2% of females reporting recent consumption. Nicotine (street name: “filong, shisha or flow”) was the second report most use drugs among secondary school students with a chi-square test showing a statistically significant difference in nicotine use between males and females students in the last 6 months (χ2 = 2.264, p = 0.322). In terms of cannabis use, the data indicated relatively low prevalence, with 2.8% of males students and 4.1% of females students reporting last 6 months use. The chi-square test did not reveal a statistically significant difference in cannabis use between males and females students in the last 6 months (χ2 = 0.595, p = 0.743) nor at any last consumption period. On the other hand, the study also examined the use of prescription medication with much information revealed on tramadol (street name: *“bagcongie”)* and diazepam (street name: ‘D10”) use. The data revealed high levels of caffeine consumption, with 82.0% of males and 88.8% of females students reporting last 6 months use, with a statistically significant difference in caffeine use between males and females students in the last 6 months (χ2 = 33.306, p < 0.001). the caffeine here includes all type of energy drinks, chocolate and kolanuts. (See Table 2)

**Table 2:** Prevalence of drug use (alterno-substance use) among study participants.

^a^Alterno-substance use (^b^Pa SU/Drug use)	Period of Use	Pool n (%)	male (n = 635) (%)	Female (n = 419) (%)	χ^2^—square	p-value
**alcohol**	Lifetime	948(89.9)	573(90.2)	375(89.5)		
	Last 6 months	784(74.4)	505(79.5)	279(66.6)	10.520	**0.005**
	Last 30 days	440(41.7)	305(48.0)	135(32.2)		
	Lifetime	191(18.1)	156(24.6)	35(8.4)		
**Nicotine**	Last 6 months	114(10.8)	90(14.2)	24(5.7)	2.264	0.322
	Last 30 days	39(3.7)	35(5.5)	4(1.0)		
	Lifetime	69(6.5)	56(8.8)	13(3.1)		
**Cannabis**	Last 6 months	30(2.8)	26(4.1)	4(1.0)	0.595	0.743
	Last 30 days	15(1.4)	13(2.0)	2(0.5)		
	Lifetime	32(3.0)	19(3.0)	13(3.1)		
**prescription medication**	Last 6 months	22(2.1)	14(2.2)	8(1.9)	0.943	0.624
	Last 30 days	5(0.5)	2(0.3)	3(0.7)		
	Lifetime	893(84.7)	521(82.0)	372(88.8)		
**Caffeine**	Last 6 months	784(74.4)	502(79.1)	282(67.3)	33.306	**<0.001**
	Last 30 days	625(59.3)	305(48.0)	320(76.4)		

^a^Alterno-substance: in the context of this work, it is a word derived from the word ’alterno’, which means an alteration or change in brain function ’psycho’ and ’substance’, typically associated with “drugs; licit and illicit’

^b^PaSU: Psychoactive substance use (drug use)

### Prevalence of global substance use (alterno-substance use)

**Fig 1:**
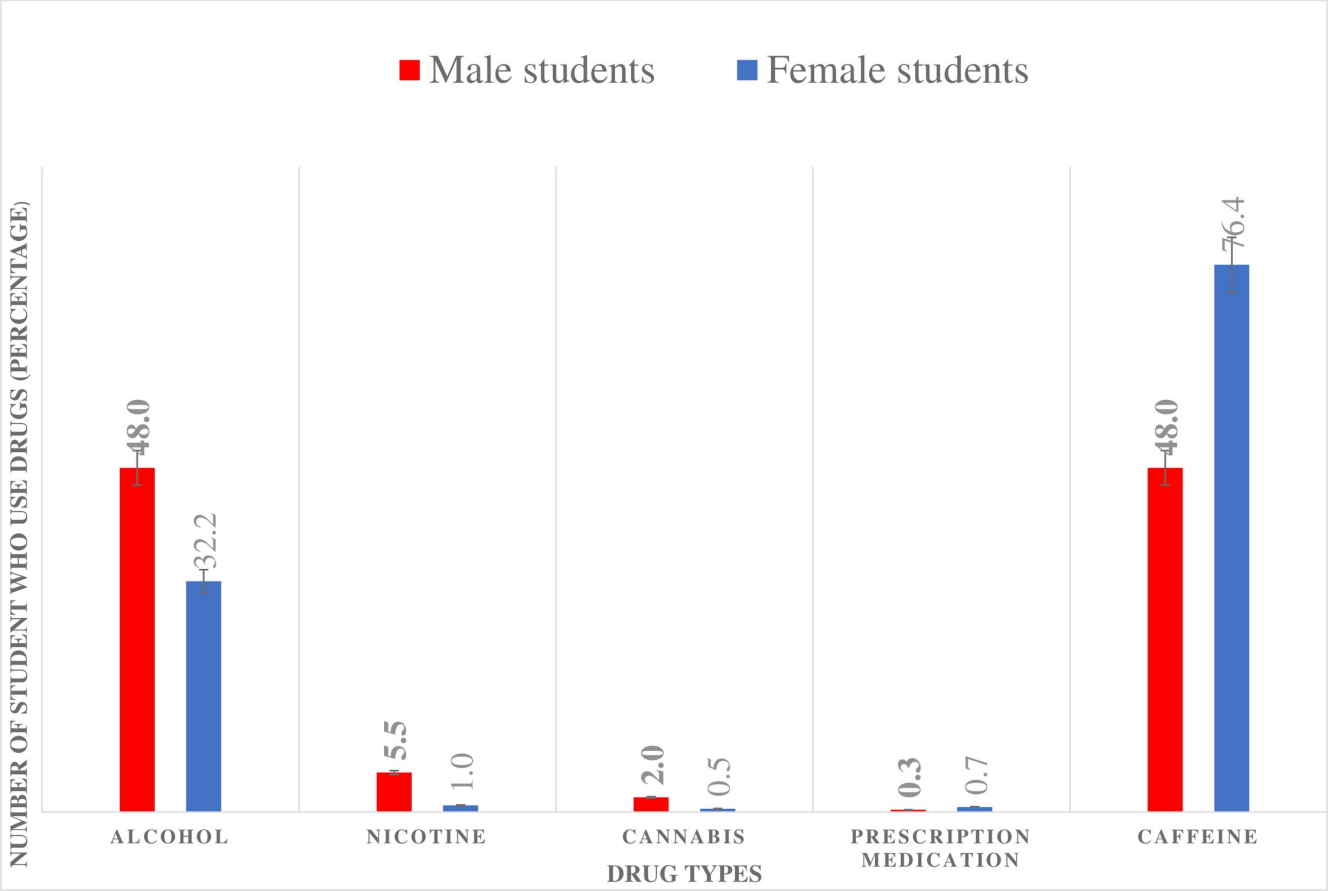
Last 30days of drug use by secondary students.

The prevalence of current drug consumption was analysed concerning sociodemographic characteristics of students. In Fig 1 it is stratified by gender, revealing a significant difference (p<0.001). Among the total sample, 49.9% of male students and 32.0% of female students reported current drug (PaSU) in the last 30 days. The difference in PaSU between genders was statistically significant, indicating a higher prevalence among male students. And more also, the age groups 14 to 17years, exhibited the highest prevalence of PaSU in the last 30 days of use.

### Motif for drug use and factors associated with drug use among students

**Fig 2:**
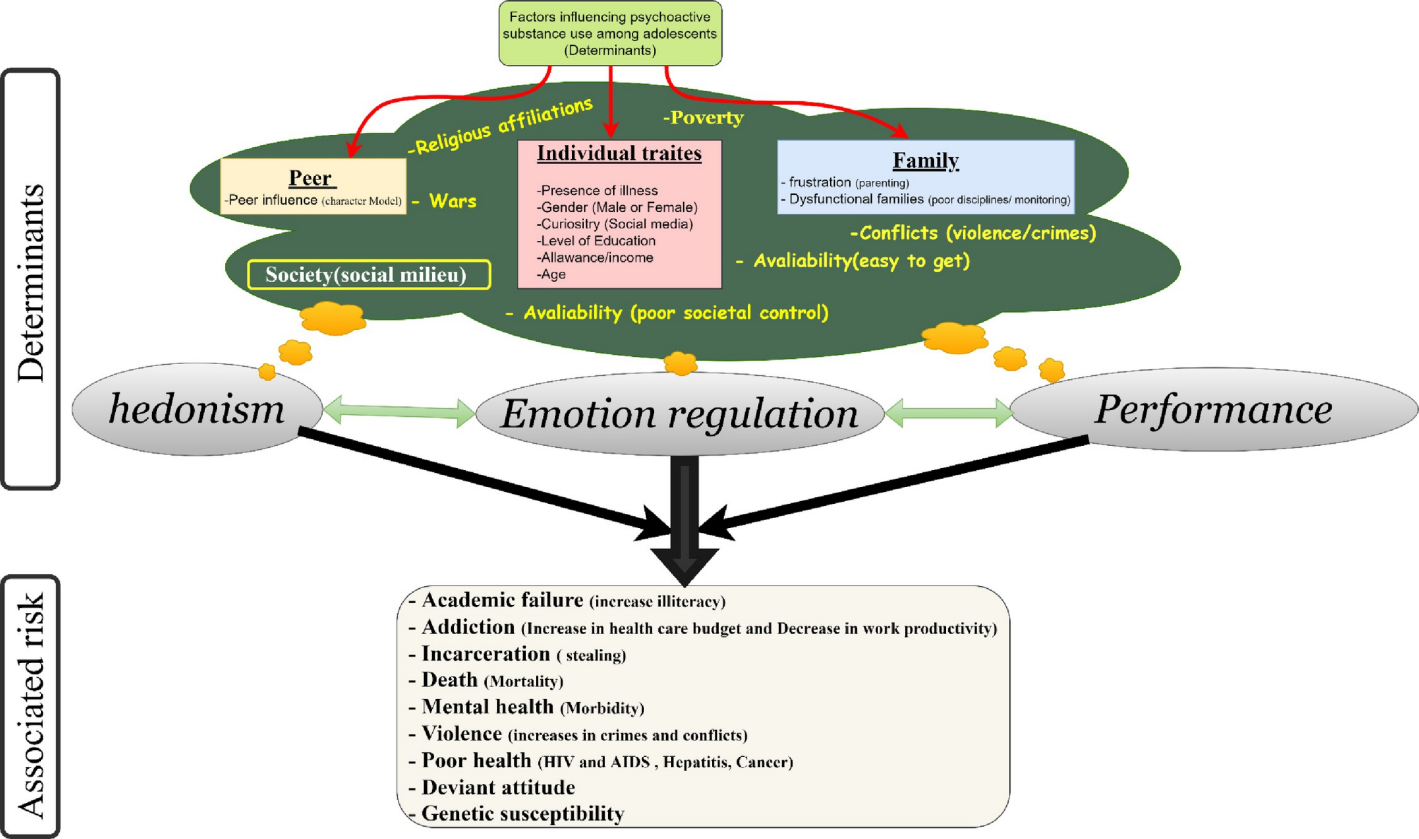
Determinants for using drugs by students (hedonism, emotion regulation and for performance).

**Fig 3:**
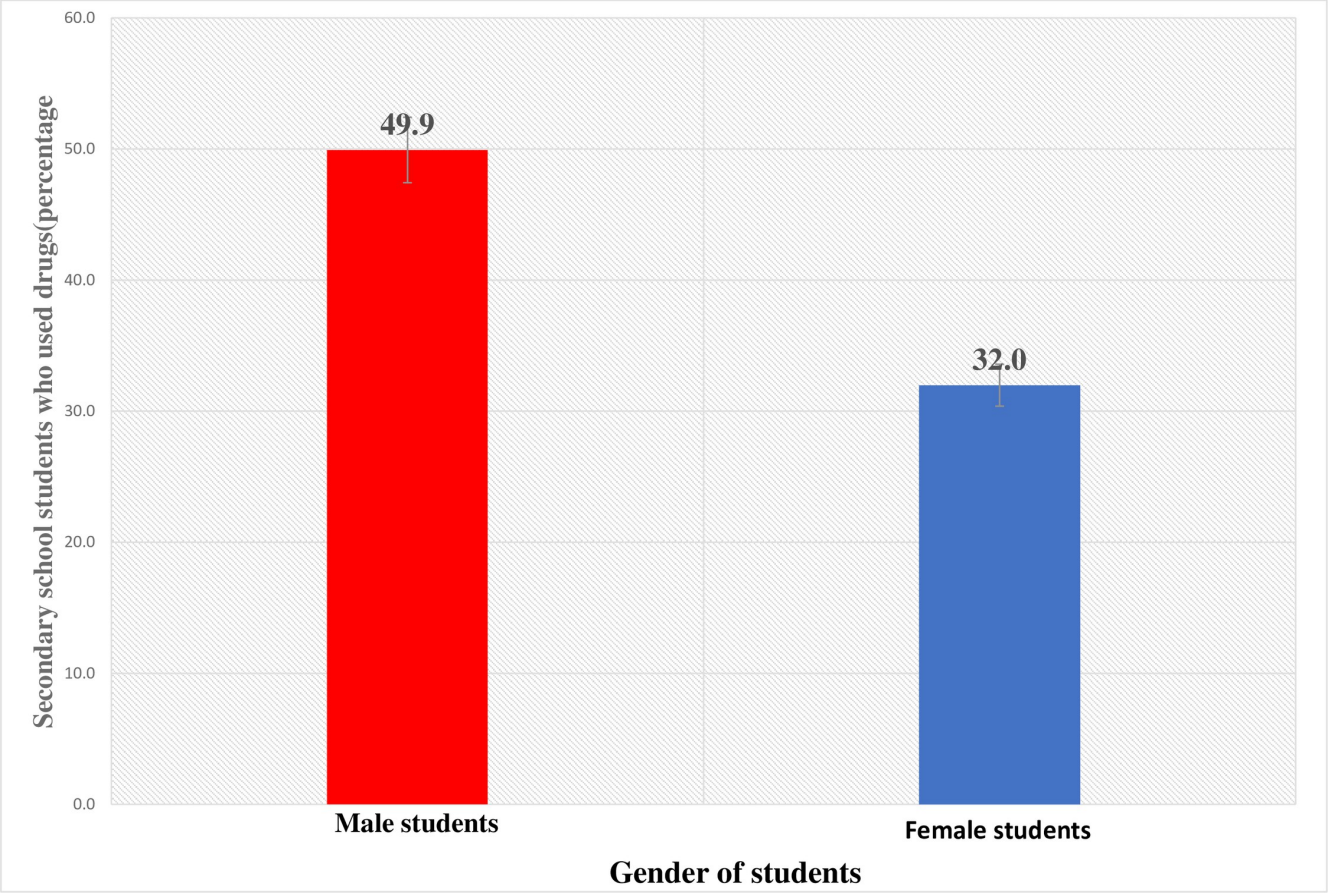
Global prevalence of drug use by students.

Several reasons were mentioned by students to justify their experimentation with psychoactive substances use (PaSU or alterno-substance), and these reasons can be grouped into three main types of motives: hedonism, emotional regulation, and performance as seen in Fig 2. In Fig 2; Hedonism, encompassing the imitation of friends, the pursuit of pleasure, the desire for new sensations, and curiosity, constituted the most prevalent motive for students’ experimentation (66.7%). The second motive was emotional regulation, with 28.3% of students having experimented with psychoactive substance (PaSU) to avoid or forget family issues (13.1%) and personal challenges (15.2%). The third motive was performance, as 5.1% of students consumed psychoactive substance (PaSU) to enhance their academic achievements. In the provided univariate analysis in Table 3 and Fig 3, several factors are examined in relation to their potential influence on a student’s decision to take drugs [7]. The factors included gender, age, daily allowance, education type, level of education, father’s drug use, mother’s education, family relative guidance, and relationships with friends [7]. The analysis presents the odds ratio (OR), 95% confidence interval (CI), and p-values for each factor. The study revealed that being a male gender (OR = 2.1; CI: 1.63–2.74; p < 0.001), age > 16 years (OR = 2.8; CI: 2.16–3.58; p < 0.001), Daily Allowance of <400frs (OR = 2.7, 95% CI = 2.61–3.76, p<0.001), attending the francophone subsystem of education (OR = 1.8; CI: 1.36–2.27.; p < 0.001), being in the second cycle that is advance level cycle of education (OR = 2.6; CI: 2.00–3.40; p < 0.001), having parents or male guidance with secondary education for fathers (OR = 1.6; CI: 1.17–2.08; p = 0.002) and primary education for mothers or female guidance (OR = 1.6; CI: 1.07–2.25; p = 0.020), the presence of a family member using drugs in the at home (OR = 2.9; CI: 2.23–3.76; p < 0.001), and having a friend using drugs (OR = 21.8; CI: 15.58–29.44; p < 0.001) were independently identified as risk factors associated with current drug consumption among secondary school students. Whereas the presence of parents with higher education: fathers (OR = 0.76; CI: 0.59–0.97; p = 0.026) and mothers with higher education (OR = 0.8; CI: 0.54–0.96; p = 0.026) constituted significant protective factors against the use of alterno-substance(drugs).

**Table 3:** Factors associated with recent drug use among secondary school students (last 30 days of drug use); univariate analysis.

Variables		Pool (n = 1054)	PaSU (n = 440) (%)	Non PaSU (n = 614) (%)	OR	95% CI	p-VALUE
	male	635	317(49.9)	318(50.1)	2.1	1.63–274	**<0.001**
**Gender**	female	419	134(32.0)	285(68.0)			
**Age**	>16years	479	269(56.2)	209(43.6)	2.8	2.16–3.58	**<0.001**
**Daily allowance**	>400	482	278(57.7)	204(42.3)	2.7	2.61–3.76	**<0.001**
	anglophone	414	143(34.5)	271(65.5)			
**education type**	francophone	640	308(48.1)	332(51.9)	1.8	1.36–2.27	**<0.001**
	ordinary level	713	251(35.2)	462(64.8)			
**level of education**	advanced level	341	200(58.7)	141(41.3)	2.6	2.00–3.41	**<0.001**
	higher education	482	191(39.6)	291(60.4)	0.8	0.59–0.97	0.26
**father (Male guidance) drug use**	secondary education	248	127(51.2)	121(48.8)	1.6	1.17–2.08	**0.002**
	primary education	43	25(58.1)	18(41.9)	1.0	0.52–1.78	0.900
	non-educated	4	1(25.0)	3(75.0)	1.8	1.66–1.85	0.140
	no idea	267	115(43.1)	152(56.9)	1.0	0.77–1.34	0.914
	higher education	258	95(36.8)	163(63.2)	0.7	0.54–0.96	**0.026**
	secondary education	433	181(41.8)	252(58.2)	0.9	1.118–2.26	0.509
**mother (female guidance) education**	primary education	128	67(52.3)	61(47.7)	1.6	1.07–2.25	**0.020**
	non-educated	61	30(49.2)	31(50.8)	1.3	0.78–2.21	0.290
	no idea	172	78(45.3)	94(54.7)	1.1	0.81–1.57	0.458
**Relationships/Neighbourhood**	family relative	545	299(54.9)	245(45.0)	2.9	2.23–3.76	**<0.001**
	friends	454	360(79.3)	94(20.7)	21.8	15.58–29.44	**<0.001**

*PaSU: Psychoactive substance use (drug use)

## Discussion

The sociodemographic distribution of the prevalence of drug use among secondary school students in this cross-sectional study reveals compelling insights. The data indicates varying rates of drug use across different time frames and substances in a diversified sample of secondary school students. For instance, the lifetime prevalence of alcohol use was notably high, with 89.9% of students reporting alcohol use, while the prevalence decreased over the last 6 months (74.4%) and the last 30 days (41.7%). Similarly, the prevalence of nicotine, cannabis, and prescription medication use followed a similar pattern, with higher rates in lifetime use compared to the last 6 months and the last 30 days. Notably, caffeine use exhibited a high prevalence across all time frames, with 74.4% reporting use in the last 6 months and 59.3% in the last 30 days. These findings highlight the pervasive nature of substance use among secondary school students, particularly for alcohol and caffeine.

Unveiling the distribution pattern of drug use among secondary school students in Douala, Cameroon, revealed interesting patterns. The study that included 1054 participants and focusing on the prevalence of drug use, particularly in the last 30 days. The data showed that 42.8% of the students reported drug use in the last 30 days, with a higher percentage among males students (49.9%) compared to females students (32.0%). Additionally, the study explored polydrug use, indicating that 69.5% of the students reported using one type of drug, with a higher percentage being among females students (76.8%) compared to males students (64.7%). The chi-square test revealed a significant association between polydrug use and gender, emphasizing the importance of considering gender-specific factors in addressing drug use among secondary school students. These findings aligned with a study conducted in 2010, reporting a higher number of males students involved in more than one substance use type, with the higher prevalence of drug use among male secondary school students [2]. Moreover, identified gender as a predictor of drug use prevalence among secondary school students, further supporting the significance of gender-specific interventions [34].

The interpretation of this data is supported by study in Nigeria on the lifetime prevalence rate of substance use among secondary students in Nigeria varied widely, aligning with the observed variations in the current study [2]. Furthermore, the study by highlighted the influence of sociodemographic factors on the prevalence of drug use, indicating that gender, educational level, and geographical economic distribution were predictors of drug use prevalence [34]. This aligns with the current findings, suggesting that sociodemographic factors may contribute to the observed variations in drug use prevalence.

Moreover, the study by emphasized the importance of monitoring problematic drug use and evaluating the necessity of prevention programs among students, which is pertinent given the high prevalence of substance use identified in the current study [35]. Additionally, the study by highlighted the association between membership in fraternity/sorority and stimulant use, indicating the influence of social factors on drug use prevalence [36].

The univariate analysis revealed several factors independently associated with current substance use disorder among students. Male gender, age over >16 years, pursuing the second cycle (educational level) of the francophone subsystem of education in Cameroon, having parents with secondary education for fathers and primary education for mothers, living with a family member who uses substances at home, and having a friend who uses substances in their social circle were identified as independent risk factors for current PaSU among secondary school students. Whereas having parents with higher education levels, both fathers and mothers, served as significant protective factors against substance use related disorder [7, 37]. The findings are consistent with existing literature on risk and protective factors for substance use among young people. Studies have shown that social influences, such as family members and peers using substances, are strong risk factors for adolescent substance use [37, 38]. Several studies, have explore the role of gender on substance use disorders with research indicating that gender differences play a significant role in the development of substance use disorders [39]

The multivariate analysis in Table 4; on risk factors associated with drug use revealed several significant associations. The analysis showed that being a male student (AOR 1.58, 95% CI 1.07–2.34, p = 0.022) and being above 16 years of age (AOR 2.94, 95% CI 2.02–4.27, p<0.001) were associated with increased odds of drug use. More also being in the francophone subsystem of education (AOR 1.71, 95% CI 1.17–2.51, p = 0.006) and being an advanced level student (second cycle) (AOR 2.46, 95% CI 1.54–3.91, p<0.001) were also associated with higher odds of drug use. The study also identify that having family relatives (AOR 3.8, 95% CI 2.61–5.53, p<0.001) and friends (AOR 32.92, 95% CI 22.02–49.20, p<0.001) in the neighbourhood/community were strongly associated with increased odds of drug use, these findings also align with other studies identifying society and relationships as a disposing factor towards drug use [40]. Additionally, the research highlighted the association between drug use and other determinants such as age and educational level, underscoring the importance of considering social and environmental factors in understanding drug use. The strong associations found with family relatives and friends in the neighbourhood/community indicate the influence of social networks on drug use behaviour [40].

**Table 4:** Multivariate analysis of factors associated with recent drug use among secondary school students (last 30 days of drug use).

Variable	AOR	95% CI	P-value
**Gender**			
male	1.58	1.07–2.34	**0.022**
**age**			
>16age	2.94	2.02–4.27	**<0.001**
**education type**			
francophone subsystem of education	1.71	1.17–2.51	**0.006**
**Level of education**			
Advance level students	2.46	1.54–3.91	**<0.001**
**parent/guidance level of education (father/male)**			
higher education	1.10	1.09–1.72	0.688
secondary	1.40	0.58–2.29	0.183
**parent/guidance level of education (mother/female)**			
higher education	0.94	0.60–1.47	0.789
primary	1.60	0.91–2.79	0.183
**Neighbourhood/community**			
family relative	3.80	2.61–5.53	**<0.001**
friends	32.92	22.02–49.20	**<0.001**

AOR: Adjusted odd ratio

## Conclusion

In conclusion, this analysis sheds light on the diverse factors influencing drug use patterns among secondary school students. The findings underscore the importance of a multifaceted approach to prevention and intervention as such, underscoring the need for tailored prevention and intervention strategies that consider gender-specific risk factors and patterns of drug use among secondary school students. By acknowledging the differences in drug use prevalence between male and female students, educational programs and support initiatives can be designed to address the specific needs of each gender, ultimately contributing to more effective and targeted interventions to reduce drug use among secondary school students in Cameroon.

### Limitations and future research directions

This cross-sectional study on drug use among students is subject to several limitations that warrant consideration. Firstly, the data collection method relied on self-assessment by the students, which introduces the potential for recall bias and underreporting of substance use. This limitation arises from the inherent subjectivity and memory limitations associated with self-reported data, which may lead to inaccuracies in the reported drug use behaviours. Furthermore, the presence of social desirability bias further compounds this limitation, as students may underreport their substance use due to societal expectations and norms. It is worth mentioning that, the generalizability of the study findings is constrained by the specific demographic and geographic focus of the sample involving adolescents from public schools in just two districts of Wouri Division of Littoral Cameroon, thereby limiting the extent to which the findings can be extrapolated to the broader youth population. By addressing these limitations, future studies can contribute to a more comprehensive understanding of drug use among educated and uneducated youth population, thereby facilitating the development of targeted interventions and policies to address this public health concern.
